# Physiological and Metabolic Responses of Marine Mussels Exposed to Toxic Cyanobacteria *Microcystis aeruginosa* and *Chrysosporum ovalisporum*

**DOI:** 10.3390/toxins12030196

**Published:** 2020-03-20

**Authors:** Flavio Oliveira, Leticia Diez-Quijada, Maria V. Turkina, João Morais, Aldo Barreiro Felpeto, Joana Azevedo, Angeles Jos, Ana M. Camean, Vitor Vasconcelos, José Carlos Martins, Alexandre Campos

**Affiliations:** 1 CIIMAR- Interdisciplinary Centre of Marine and Environmental Research, University of Porto, Terminal de Cruzeiros do Porto de Leixões, Av. General Norton de Matos, s/n, 4450–208 Porto, Portugal; up201510053@fc.up.pt (F.O.); jmorais@ciimar.up.pt (J.M.); aldo.barreiro@gmail.com (A.B.F.); joana_passo@hotmail.com (J.A.); vmvascon@fc.up.pt (V.V.); jmartins@ciimar.up.pt (J.C.M.); 2 Area of Toxicology, Faculty of Pharmacy, Universidad de Sevilla, Profesor García González n2, 41012 Seville, Spain; ldiezquijada@us.es (L.D.-Q.); angelesjos@us.es (A.J.); camean@us.es (A.M.C.); 3 Department of Biomedical and Clinical Sciences, Faculty of Medicine and Clinical Sciences, Linköping University, 581 83 Linköping, Sweden; maria.turkina@liu.se; 4 Biology Department, Faculty of Sciences, University of Porto, Rua do Campo Alegre, s/n, 4169–007 Porto, Portugal

**Keywords:** *Mytilus galloprovincialis*, toxic cyanobacteria, microcystin, cylindrospermopsin, ecotoxicology, shotgun proteomics

## Abstract

Toxic cyanobacterial blooms are a major contaminant in inland aquatic ecosystems. Furthermore, toxic blooms are carried downstream by rivers and waterways to estuarine and coastal ecosystems. Concerning marine and estuarine animal species, very little is known about how these species are affected by the exposure to freshwater cyanobacteria and cyanotoxins. So far, most of the knowledge has been gathered from freshwater bivalve molluscs. This work aimed to infer the sensitivity of the marine mussel *Mytilus galloprovincialis* to single as well as mixed toxic cyanobacterial cultures and the underlying molecular responses mediated by toxic cyanobacteria. For this purpose, a mussel exposure experiment was outlined with two toxic cyanobacteria species, *Microcystis aeruginosa* and *Chrysosporum ovalisporum* at 1 × 10^5^ cells/mL, resembling a natural cyanobacteria bloom. The estimated amount of toxins produced by *M. aeruginosa* and *C. ovalisporum* were respectively 0.023 pg/cell of microcystin-LR (MC-LR) and 7.854 pg/cell of cylindrospermopsin (CYN). After 15 days of exposure to single and mixed cyanobacteria, a depuration phase followed, during which mussels were fed only non-toxic microalga *Parachlorella kessleri*. The results showed that the marine mussel is able to filter toxic cyanobacteria at a rate equal or higher than the non-toxic microalga *P. kessleri*. Filtration rates observed after 15 days of feeding toxic microalgae were 1773.04 mL/ind.h (for *M. aeruginosa*), 2151.83 mL/ind.h (for *C. ovalisporum*), 1673.29 mL/ind.h (for the mixture of the 2 cyanobacteria) and 2539.25 mL/ind.h (for the non-toxic *P. kessleri*). Filtering toxic microalgae in combination resulted in the accumulation of 14.17 ng/g dw MC-LR and 92.08 ng/g dw CYN. Other physiological and biochemical endpoints (dry weight, byssus production, total protein and glycogen) measured in this work did not change significantly in the groups exposed to toxic cyanobacteria with regard to control group, suggesting that mussels were not affected with the toxic microalgae. Nevertheless, proteomics revealed changes in metabolism of mussels related to diet, specially evident in those fed on combined cyanobacteria. Changes in metabolic pathways related with protein folding and stabilization, cytoskeleton structure, and gene transcription/translation were observed after exposure and feeding toxic cyanobacteria. These changes occur in vital metabolic processes and may contribute to protect mussels from toxic effects of the toxins MC-LR and CYN.

## 1. Introduction

Cyanobacterial blooms are among the main causes of contamination of freshwater ecosystems. Furthermore major adverse effects in the aquatic ecosystems and human health, caused by the presence and exposure to toxic cyanobacteria and cyanotoxins, have been studied and identified. The “blooms” of cyanobacteria are known to impair water quality, for instance by reducing oxygen in the water and light [1,2,3]. On the other hand the toxic effects of cyanotoxins on organisms may impair aquatic biodiversity [3]. Concerning human health, the presence of toxic cyanobacteria and cyanotoxins in drinking waters, recreational waters, and in food increases the risk of exposure, toxin poisoning, and the development of illnesses.

Among the toxins produced by cyanobacteria in freshwaters are microcystins (MCs), cylinderspermopsins (CYN) and saxitoxins (STXs). MCs comprise a broad group of cyclic heptapeptides with the amino acid sequence D-alanine-X-DMeAsp-Z-Adda-D-glutamate-Mdha, in which X and Z are variable L-amino acids (molecular formula C_49_H_74_N_10_O_12_). MCs are produced by species from the genera *Microcystis*, *Anabaena*, *Anabaenopsis*, *Aphanizomenon*, *Limnothrix*, *Nostoc*, *Oscillatoria*, *Phormidium*, *Planktothrix*, *Woronichinia*, and are the most prevalent group of toxins in freshwater ecosystems. Also MCs are considered the most potent and adverse group of cyanotoxins [4,5]. CYN is a cyclic alkaloid with the molecular formula C_15_H_21_N_5_O_7_S [6,7], and is produced by species of the genera *Chrysosporum*, *Anabaena*, *Raphidopsis*, and *Umezakia*. A significant dispersion of CYN from tropical to temperate regions of the world have been documented, possibly due to changes in the ecosystems derived from global warming [8,9]. This worldwide dispersion of CYN poses new risks to aquatic ecosystems and human health. Although considered one of the most relevant cyanotoxins from freshwaters and most detrimental to humans, CYN is not as well understood as MCs, concerning the mode of action and mechanisms of toxicity [10]. Research has provided evidences that this toxin inhibit protein and glutathione (GSH) biosynthesis [11] and thereby these are among the most relevant toxicity mechanisms associated with the toxin.

In freshwater ecosystems, bivalves have shown to be able to filter and feed on cyanobacteria and to accumulate toxins such as MCs and CYN [12,13,14]. Alongside, ingestion and assimilation of toxic cyanobacteria can cause several physiological and metabolic responses and may affect animal scope for growth [15], bioenergetics [16] and animal immune system [17,18]. At the molecular level, alterations in cytoskeleton proteins, in energy metabolism, and activation of xenobiotic metabolism enzymes have been reported in bivalves exposed to toxic cyanobacteria and cyanotoxins [19,20,21]. Moreover bivalve molluscs have been shown to retain MCs in the tissues for long periods and asymptomatic toxin clearance kinetics [14]. MCs although being highly toxic substances to many organims, bivalves have been shown to tolerate the accumulation and potential adverse effects of these toxins [20,22].

Regarding estuarine and marine bivalve species, a question still remains whether these exhibit the same behavior and tolerance as their freshwater relatives, to feeding on toxic cyanobacteria and exposure to cyanotoxins. Studies have shown that estuarine and marine bivalve species such as *Mytilus galloprovincialis* and *Ruditapes philippinarum* modulate their detoxification and oxidative-stress defense systems after exposure to MCs [23,24]. Indeed, contamination of estuarine and coastal areas derived from the flow of contaminated freshwaters from inland to the sea have been reported in different geographical regions [25,26,27], constituting a potential thread to marine ecosystems. For example, high levels of MCs were found in mussels collected from San Francisco Bay, California [26]. Moreover, a monitoring study in this estuary showed that the contamination of bivalves with MCs is recurrent being detected in several years of the monitoring study [27]. Another monitoring investigation revealed a significant transfer of cyanobacteria and cyanotoxins along a freshwater-marine continuum in France. The authors reported that salinity is the main factor determining the spatial variation of cyanobacteria. Moreover several *Microcystis* species showed to resist intermediate salinities in this freshwater-marine continuum. At the most downstream estuarine site MCs were detected mostly dissolved in the water, consistent with the increased cell lysis caused by high sanility [28]. Kim et al. (2019) [29] reported great amounts of MCs (concentrations varying from 0.4 to 75 μgL^−1^) in Geum River Estuary, Korea, associated with increased freshwater discharges during the rainy season. Accumulation of MCs was reported in several marine organisms, but was higher in organisms of lower trophic positions. The fatality of sea otters associated with MCs poisoning [30] well reveals the extension of the impacts one can expect from the presence of cyanotoxins in marine ecosystems. Moreover recent research revealed that Microcystis strains developed several mechanisms that enable toxic and non-toxic strains to survive in salinity environments [31].

In line with the evidences from freshwater cyanobacteria occurrence and persistence in brakish and marine environments, this research work aimed to investigate the sensitivity of marine mussels (*M. galloprovincialis*) to single and mixed cultures of two toxic cyanobacterial species from freshwaters. To this purpose, a natural condition of exposure of mussels to toxic cyanobacteria was reproduced in the laboratory, with the MCs producer cyanobacteria *Microcystis aeruginosa*, and the CYN producer species *Chrysosporum ovalisporum*. *Parachlorella kessleri* was the non-toxic microalga in the experiment. The response of mussels to exposure to toxic cyanobacteria was investigated considering several physiological and biochemical end-points and proteomic analysis.

## 2. Results

### 2.1. Physiological Parameters

**Figure 1:**
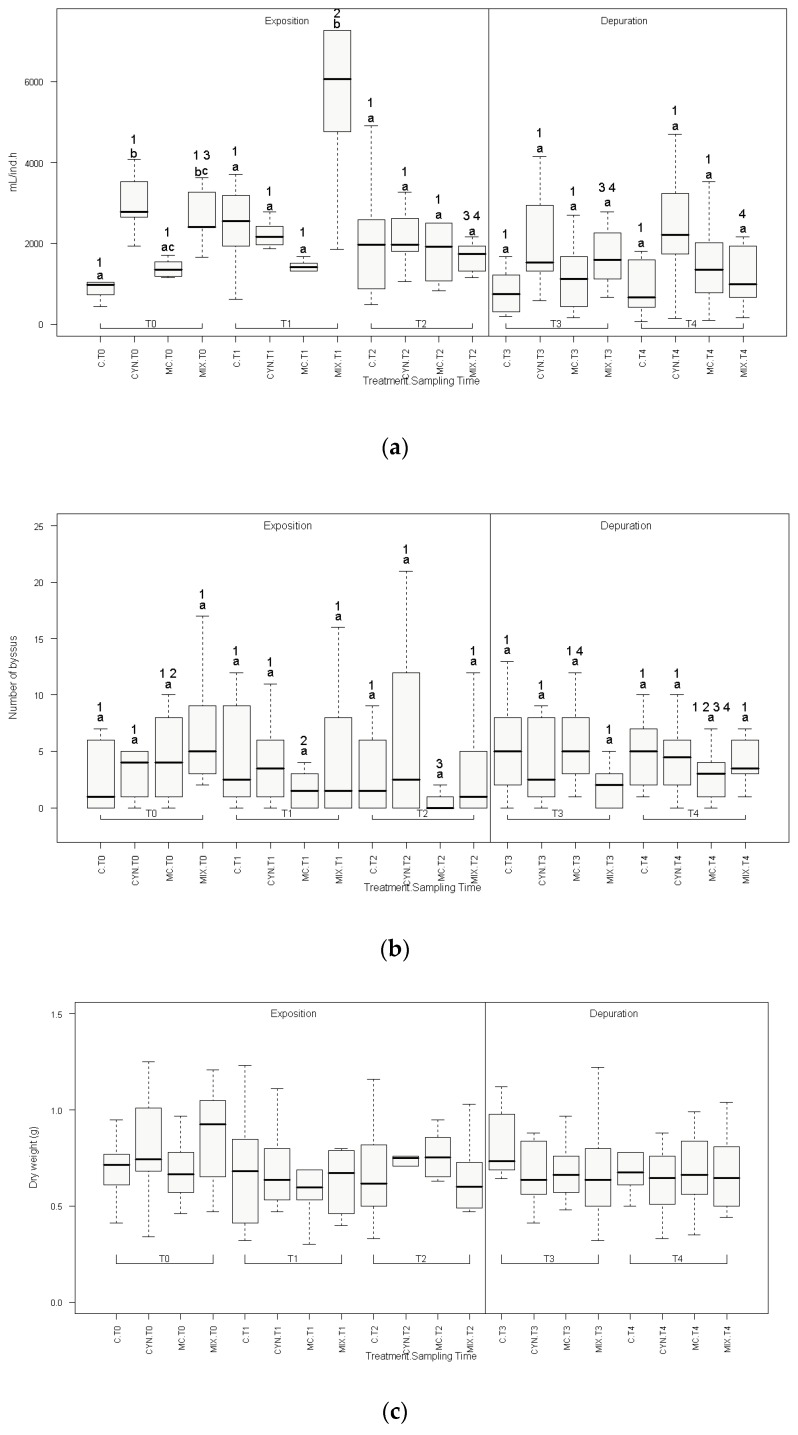
Mussels filtration rates (**a**), number of byssal threads (**b**) and whole body dry weight (**c**) during the exposition and recovery/depuration phases of the study. (C) Control group; (CYN) group exposed to *C. ovalisporum* cells; (MC) group exposed to *M. aeruginosa* cells; (MIX) group exposed to *C. ovalisporum* and *M. aeruginosa* cells. Time of the experiment in weeks (T0; T1; T2; T3; T4). Different letters indicate statistical differences (*p* < 0.05) between treatments, for each sampling time. Different numbers indicate statistical differences (*p* < 0.05) related with time, in each experimental group (*n* = 10). These differences were tested with a Student *t*-test for dependent samples, correcting critical *p* values for multiple comparisons with Bonferroni correction.

The filtration rates were measured in the begginig of the experiment (T0) and then every week, during the exposition phase of two weeks (T1, T2) and the depuration phase also with a duration of two weeks (T3 and T4). The results are shown in Figure 1a and the corresponding statistical analysis in Supplementary Tables S1–S3. Initial measurements (T0) denoted significantly higher filtration rates for *C. ovalisporum* cells (CYN group) and cyanobacteria mixture (MIX group) in comparison with the non-toxic microalga *P. kessleri* (C group) (2.8 and 2.6-fold, respectively, *p* < 0.05). After one week (T1), mussels exposed to the cyanobacteria mixture (CYN group) continued to show significantly higher filtration rates comparatively to the control group (2.2-fold, *p* < 0.05). However at the end of the exposition phase (T2) and during depuration (T3, T4) no differences were found in the filtration rates of toxic cyanobacteria provided in separate or in mixture to mussels with regard to *P. kessleri* filtration rate (C group). During the depuration phase mussels from all experimental groups were exposed only the non-toxic alga *P. kessleri*. In this period, filtration tests with toxic microalgae showed that mussels previously feeding on *C. ovalisporum*, *M. aeruginosa*, or with the mixture of the 2 cyanobacteria continued to be able to filter and feed on the toxic algae, showing filtering patterns between treatments similar to those initially found (T0). It should be noted also that pseudofaeces were not observed during mussel grazing in toxic cyanobacteria.

No significant effects of treatment globally were observed in the production of byssal threads (Figure 1b, Supplementary Table S1) and in the whole body dry weight (Figure 1c) between experimental groups along the entire length of the experiment (exposition and depuration phases).

### 2.2. Biochemical Parameters

**Figure 2:**
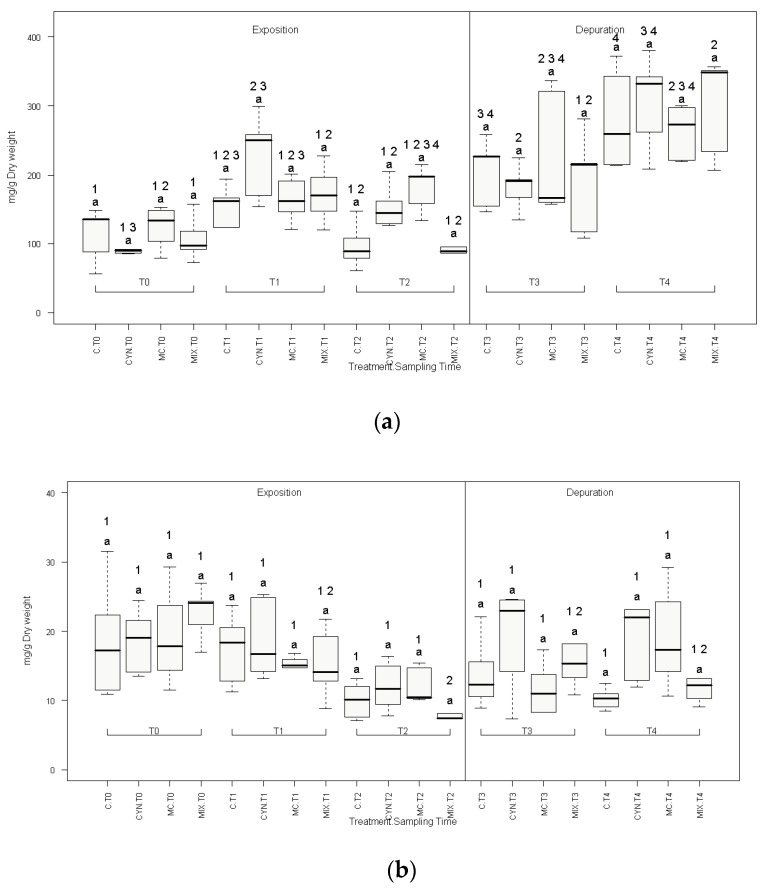
Total protein (**a**) and glycogen (**b**) variations in mussel’s whole body during the exposition and recovery/depuration phases of the study. (C) Control group; (CYN) group exposed to *C. ovalisporum* cells; (MC) group exposed to *M. aeruginosa* cells; (MIX) group exposed to *C. ovalisporum* and *M. aeruginosa* cells. Time of the experiment in weeks (T0; T1; T2; T3; T4). Different letters indicate statistical differences (*p* < 0.05) between treatments, for each sampling time. Different numbers indicate statistical differences (*p* < 0.05) related with time, in each experimental group (*n* = 5). These differences were tested with a Student *t*-test for dependent samples, correcting critical *p* values for multiple comparisons with Bonferroni correction.

Total protein content (protein amount per body weight) in mussels was not significantly affected globally by treatment (Figure 2a, Supplementary Tables S1 and S3), during both experimental phases (exposition and depuration), as shown in Figure 2a. Nonetheless, total protein increased gradually in all experimental groups along the experiment. Significant increases in total protein were verified during depuration comparatively with the exposition phase. The highest values were observed at the end of the depuration (T4), in all experimental groups (Figure 2a).

Similarly, total glycogen (glycogen amount per body weight) was not affected globally by treatment, however a slight descrease was observed in all experimental groups (including control) at the end of the intoxication phase (T2) (Figure 2b, Supplementary Tables S1 and S3).

### 2.3. Toxin Accumulation in Mussels

**Figure 3:**
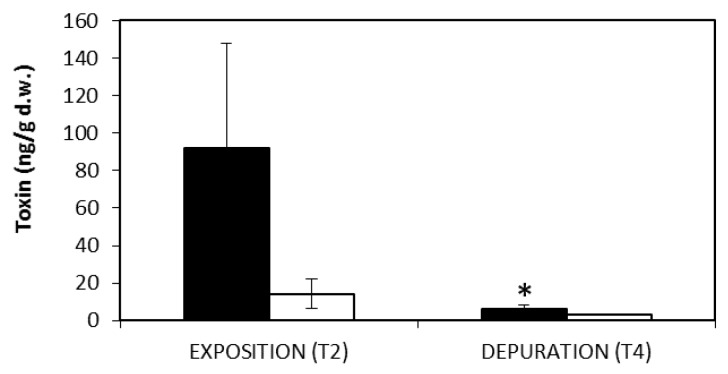
Levels of CYN and MC-LR detected in mussels whole body at the end of the exposition and the depuration phases of the experiment. Mean and standard deviation values (*n* = 3) are represented in columns (white—MC-LR and black—CYN) and bars, respectively. Significant *t*-test comparisons of toxin concentrations between exposition and depuration phases (*p* < 0.05) (*).

The accumulation of toxins CYN and MC-LR in mussels was investigated in the experimental group exposed simultaneously to the 2 toxic cyanobacteria (MIX group). Indeed, the filtration rates of microalgae observed in mussels from MIX group strongly suggested the ingestion of both types of cyanobacterial cells by mussels and to the hypothesis that the bivalves could be contaminated with both toxins. The results show that mussels accumulated respectively 14.17 ng/g dry weight (DW) MC-LR and 92.08 ng/g DW CYN during the exposition phase (Figure 3). The higher amounts of CYN observed in mussel tissues relatively to MC-LR, is in agreement with the higher amount of toxin produced by *C. ovalisporum* cells (7.850 pg/cell CYN) comparatively to *M. aeruginosa* cells (0.033 pg/cell MC-LR). After 15 days of recovery, the content of CYN in mussels decreased significantly (*p* < 0.05) to 7% of the content observed at the end of the exposition phase. Mussels contaminated with MC-LR also eliminated part of the toxin during the depuration phase, nevertheless the decrease was not statistically significant (Figure 3).

### 2.4. Proteomics

**Figure 4:**
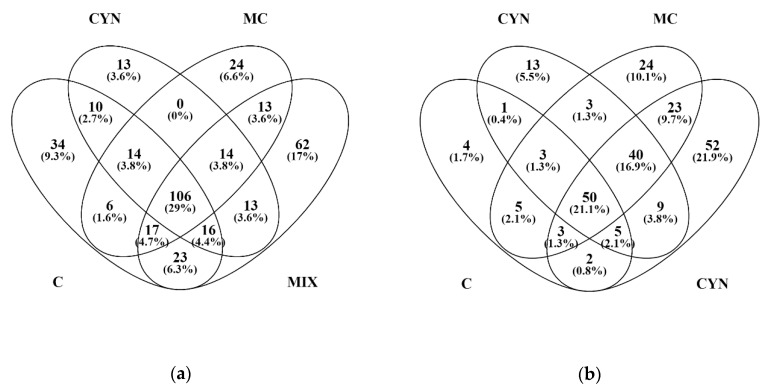
Venn diagram of the total number proteins identified, in the different experimental conditions, at the end of exposition (**a**) and depuration (**b**) phases of the study. (C) Control group; (CYN) group exposed to *C. ovalisporum* cells; (MC) group exposed to *M. aeruginosa* cells; (MIX) group exposed to *C. ovalisporum* and *M. aeruginosa* cells.

A quantitative shotgun proteomics analysis was carried out to investigate the molecular responses of mussels to the exposure and feeding with toxic cyanobacteria. Following a procedure adapted from Campos et al. (2016) [32], we were able to identify in total 394 proteins from the digestive gland of all samples analysed and determine the relative expression of this set of proteins in each experimental group. Most of the proteins were identified in more than one experimental condition as shown in Figure 4. The exclusive proteins, i.e., proteins identified exclusively in each experimental condition, represented 13% and 17% of total proteins identified respectively at T2 (end of exposition) and T4 (end of the depuration) (Figure 4). Moreover, MIX was the experimental group where more exclusive proteins were identified, at T2 and also at T4.

**Figure 5:**
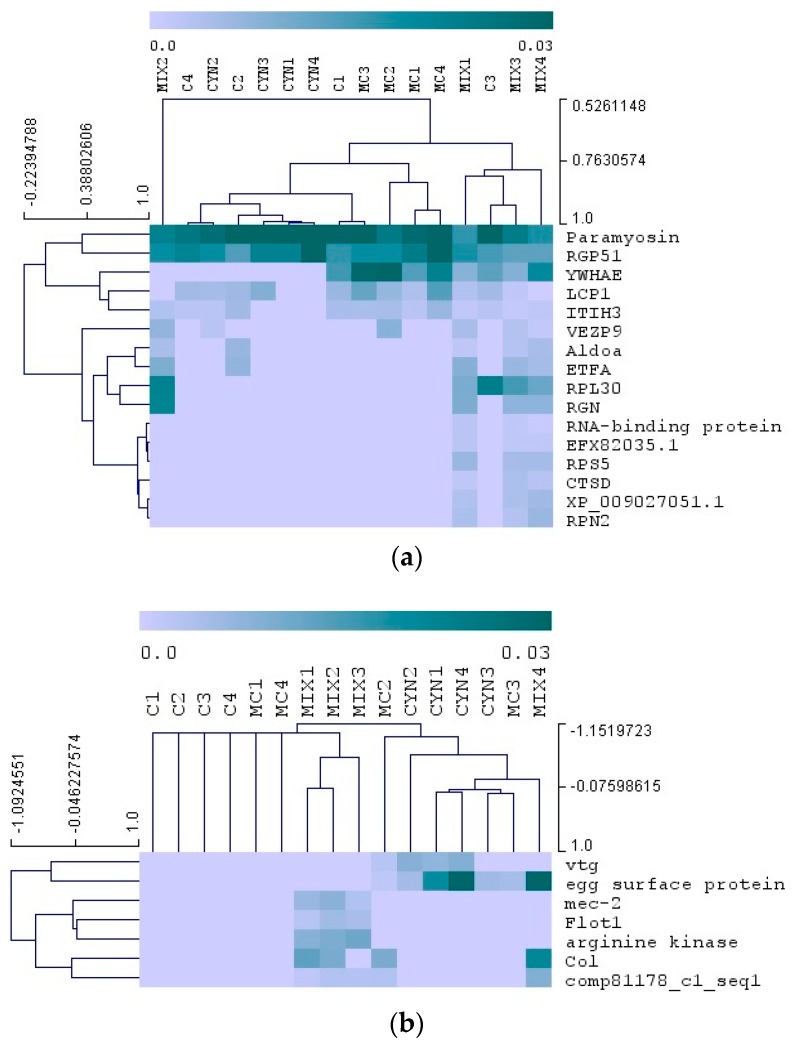
Multivariate analysis of mussel proteins after animal exposure (T2) to toxic cyanobacteria (**a**) and after depuration (T4) (**b**). The color map represents the relative expression of differential proteins (Kruskal-wallis test, *p* < 0.05). Proteins are reported in lines and group samples (4 replicates, *n* = 4) in columns. Control group (C); group exposed to *C. ovalisporum* cells (CYN); group exposed to *M. aeruginosa* cells (MC); group exposed to *C. ovalisporum* and *M. aeruginosa* cells (MIX). Full protein names and expression levels are reported in Supplementary Table S4.

A multivariate analysis (Kruskal–Wallis test) was carried out to compare the proteomes of mussels exposed to the toxic cyanobacteria, and after depuration. As shown in Figure 5, mussel exposure and feeding on toxic cyanobacteria resulted in significant variations in the expression of several groups of proteins (Figure 5a). On the other hand, after depuration less protein expression differences were observed between experimental groups (Figure 5b). Expression levels of each protein and significance of the statistical tests are reported in Supplementary Table S4.

Furthermore, the relative expression levels of proteins were analyzed by hierarchical clustering. This analysis rendered incomplete grouping of sample replicates of each experimental group, in both experimental phases (exposition and depuration) (Figure 5). This result could reflect the highly variable expression of some proteins within replicates in, at least, some experimental groups. Among the proteins altered during the exposition of mussels with toxic cyanobacteria are those with putative functions in signaling and communication (retrograde protein of 51 kDa—RGP51), cell structure and movement (paramyosin, plastin-2—LCP1), regulation of protein activity (14-3-3 protein epsilon—YWHAE, dolichyl-diphosphooligosaccharide--protein glycosyltransferase—RPN2, cathepsin D—CTSD), cell proliferation and migration (inter-alpha-trypsin inhibitor heavy chain H3—ITIH3), germ cell functions (vitelline envelope zona pellucida domain 9—VEZP9), energy metabolism (fructose-bisphosphate aldolase—Aldoa, electron transfer flavoprotein subunit alpha—ETFA), gene transcription/translation (ribosomal protein L30—RPL30, 40S ribosomal protein S5—RPS5, RNA-binding protein), and cellular calcium ion homeostasis (regucalcin—RGN). Functions of 2 differentially expressed proteins could not be assigned given that presented homology with protein sequences from National Center for Biotechnology Information (NCBI) lacking functional annotation (EFX82035.1, XP_009027051.1). Within the proteins with altered expression at the end of the depuration phase, some may be associated to embryogenesis (vitellogenin—vtg, egg surface protein), and others to extracellular matrix structure (collagen-like protein-2—Col), signaling and communication (mechanosensory protein 2—mec-2), endocytosis (flotillin-1—Flot1) and regulation of protein activity (arginine kinase). One differentially expressed protein (comp81178_c1_seq) lacked homology with protein sequences from NCBI thereby its function was not assigned.

**Figure 6:**
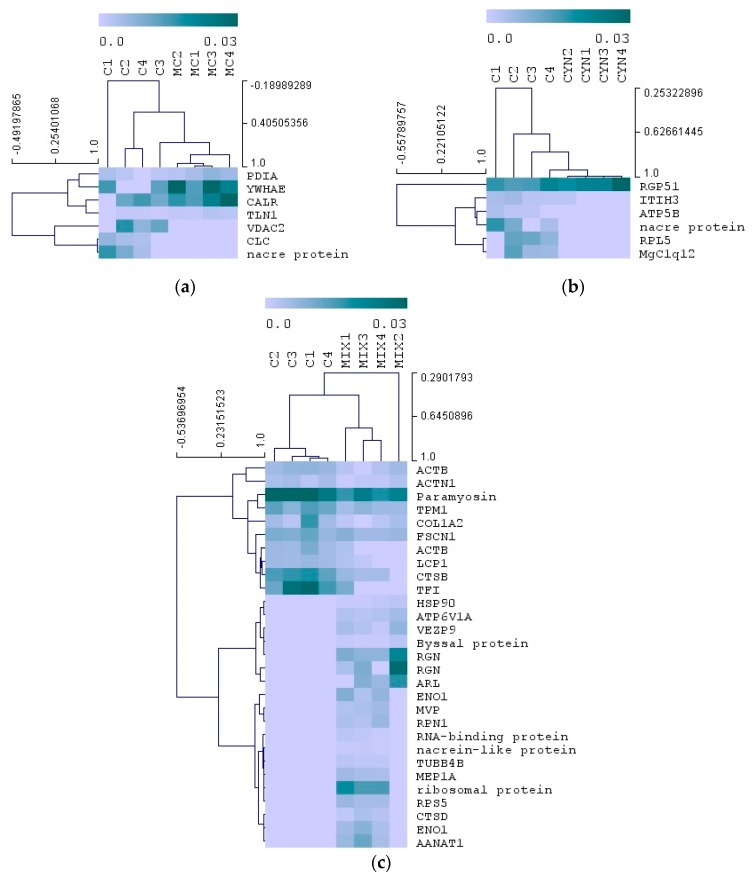
Pairwise analysis (Wilcoxon test) of mussel proteins after animal exposure to toxic cyanobacteria (exposition phase—T2). The color map represents the relative expression of differential proteins (*p* < 0.05). Proteins are reported in lines and group samples (4 replicates, *n* = 4) in columns. Comparisons between control mussels and mussels exposed to *M. aeruginosa* cells—MC (**a**); to *C. ovalisporum* cells—CYN (**b**) and to the mixture of *C. ovalisporum* and *M. aeruginosa* cells—MIX (**c**). Full protein names and expression levels are reported in Supplementary Table S5.

A pairwise (Wilcoxon test) analysis was carried out with the aim to reveal proteome alterations between control and the other experimental groups during the exposition phase, as this was the most potentially detrimental phase to mussels. The analysis revealed differential expressions in 7 and 6 proteins, in mussels exposed to *M. aeruginosa* and *C. ovalisporum* cells respectively (Figure 6a,b). Moreover, the exposure of mussels simultaneously to the two toxic cyanobacteria led to alterations in the expression of 29 proteins (Figure 6c). Expression levels of each protein and significance of the statistical tests are reported in Supplementary Table S5. The proteins altered specifically by *C. ovalisporum* were identified as RGP51, ATP synthase beta subunit (ATP5B), ITIH3, 60S ribosomal protein L5 (RPL5), nacre protein and C1q domain containing protein (MgC1q12) (Figure 6a).

Proteins altered specifically by the exposure to toxic *M. aeruginosa* were identified as protein disulfide-isomerase (PDIA), calreticulin (CALR), YWHAE, voltage-dependent anion channel 2 (VDAC2), galectin (CLC), talin-1 (TLN1), and nacre protein (Figure 6b).

The exposure to the two toxic cyanobacteria led to more alterations in the proteome of mussels, comparatively to the other two experimental conditions (Figure 6c). Among the proteins that displayed significant changes in expression are structural proteins and proteins associated to cytoskeleton (actin —ACTB, paramyosin, tropomyosin—TPM1, alpha actinin—ACTN1, collagen alpha-2(I) chain—COL1A2, LCP1, fascin—FSCN1, tubulin beta-4B chain—TUBB4B), and proteins with a possible role in regulation of protein activity (heat shock protein 90—HSP90, dolichyl-diphosphooligosaccharide--protein glycosyltransferase—RPN1). Moreover, other proteins affected by this treatment are cathepsin B (CTSB), meprin A subunit alpha (MEP1A) and cathepsin D (CTSD), which function has been associated with protein catabolism. Proteins that participate in the metabolism of energy (enolase—ENO1), and proteins with functions related with gene transcription/translation (ribosomal protein, RNA-binding protein, RPS5), cellular calcium ion homeostasis (RGN), cellular transport (ADP-ribosylation factor—ARL, major vault protein—MVP, V-type proton ATPase catalytic subunit A—ATP6V1A), melatonin biosynthesis (dopamine N-acetyltransferase—AANAT1), shell structure (nacrein-like protein), mussel adhesion (byssal protein), digestive gland functions (trefoil factor—TFI), and oocyte functions (VEZP9) were also altered by this treatment.

**Figure 7:**
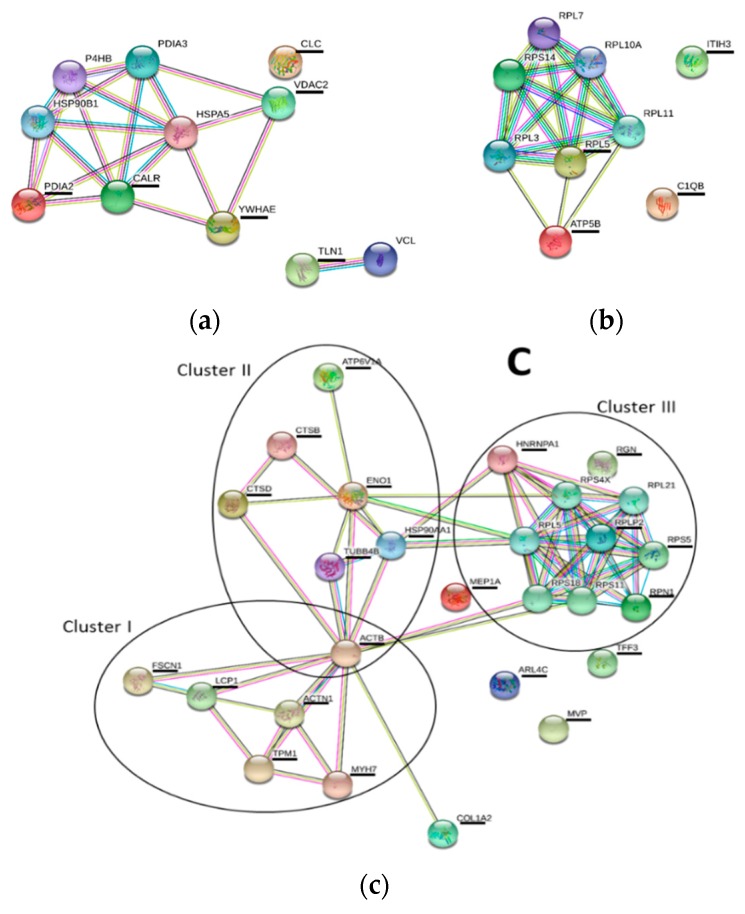
Predicted protein functional associations, from STRING database analysis. Proteins identified in this work are underlined whereas other proteins displayed in the network are potential interactors predicted by the program. The evidences supporting these associations come from different sources (curated databases, experimentally determined, gene neighborhood, gene fusions, gene co-occurrence, textmining, co-expression, protein homology) and are represented by edges of different colors (for detailed legend of the edges, consult the program page https://string-db.org/). Mussels exposed to *M. aeruginosa* cells—MC (**a**); mussels exposed to *C. ovalisporum* cells—CYN (**b**); mussels exposed to *C. ovalisporum* and *M. aeruginosa* cells—MIX (**c**).

Functional analysis of the differential proteins, using the bioinformatics program STRING (https://string-db.org/) (Figure 7) was subsequently performed in order to gather more insights concerning the functional relationships between proteins. The analysis was performed based on the information available from human homologous proteins. Proteins that were not included in this analysis, since no human homologs were found, were nacre protein, RGP51, byssal protein, AANAT1, and VEZP9. Furthermore mussel proteins such as MgCLq12, paramyosin, ribosomal protein, RNA binding protein, ARL, TFI showed homology respectively with human C1qB (complement C1q subcomponent subunit B), MYH7 (myosin-7), RPLP2 (60S acidic ribosomal protein P2), HNRNPA1 (heterogeneous nuclear ribonucleoprotein A1), ARL4C (ADP-ribosylation factor-like protein 4C) and TFF3 (trefoil factor 3). As shown in Figure 7, interactions between differentially expressed proteins were found. For instance, VDAC2, YWHAE, CALR and PDIA, identified in this work after exposure of mussels to *M. aeruginosa* cells, all take part in a protein network in which the protein HSPA5 (not identified in this work) is the central element of the network (Figure 7a). In contrast, few functional links seem to exist in the proteins altered in mussels exposed to *C. ovalisporum* cells. Indeed only a functional link between RPL5 and ATP5B was inferred in this analysis (Figure 7b). Concerning the differentially expressed proteins in mussels exposed to both cyanobacteria, these may be also related in the functions they participate, as shown in STRING analysis (Figure 7c). Within this interaction network, at least three protein clusters could be distinguished (delimited in circles, Figure 7c), i.e., the cluster I that includes the cytoskeletal and associated proteins (ACTB, MYH7, TPM1, ACTN1, LCP1, FSCN1), the cluster II with ENO1 and ACTB (ACTB, TUBB4B, HSP90AA1, ENO1, CTSD, CTSB, ATP6V1A), and the cluster III that includes the ribosomal proteins (RGN, RPLP2, RPS5, RPN1, HNRNPA1).

## 3. Discussion

### 3.1. Implication of Cyanobacteria Feeding in Mussels Health

Bivalves, including mussels, feed by filtering water and selecting small sized <100 µm nutrient particles [33]. Bivalve grazing is often dictated by the size of particles and phytoplankton cells, nevertheless some differences related with the quality of the food and the content in polyunsaturated fatty acid (PUFAs) and toxins will determine which food particles will be assimilated by bivalves. On the other hand, it was demonstrated, in field and laboratory studies, that bivalves are capable of feeding on toxic cyanobacteria [34,35]. Yet, it is not well-established which cyanobacteria species can be selected as diet and the ones which are rejected by bivalves. For instance, it was observed by Cataldo et al. (2012) [36] that the freshwater mussel *Limnoperna fortunei* is able to feed on single cells and small colonies of *Microcystis* spp. but tend to reject the large size colonies. In fact, *L. fortunei* promoted aggregation of solitary *Microcystis* spp. cells into colonies. From the ecological point of view, this selective grazing has significant impact on the phytoplankton structure and could lead to enhanced growth of *Microcystis* spp. over other phytoplankton species. In another study, Gazulha et al. (2012) [37] highlighted the feeding of the same mussel on toxic *Microcystis*. The authors pointed out that cyanobacteria toxicity was not the main factor influencing *L. fortunei* feeding behavior. Indeed this mussel species showed similar filtration rates for toxic (MC producing) and non-toxic *Microcystis* strains, in 5-day feeding experiments. Additionally, short-term experiments evidenced preferential ingestion of both toxic and non-toxic *Microcystis* cells over the non-toxic phytoplankton species *Nitzschia palea*. No physiological stress or mortality was reported in *L. fortunei* fed on toxic cyanobacteria. On the other hand, and according to Juhel et al. (2006) [15], the feeding on toxic cyanobacteria can trigger sublethal, stressful effects in zebra mussels (*Dreissena polymorpha*). The feeding behavior and energy balance of zebra mussels on five microalgal diets, including two toxic *M. aeruginosa* strains, was studied at comparable masses of suspended matter (45 mg/L) in a flow-through system. This study showed that the cyanobacterium strain producing the toxin MC-LF is cleared and ingested by mussels at significant lower rate comparatively to all other phytoplankton species studied including the cyanobacterium producing MC-LR. Another response observed in zebra mussels fed on toxic cyanobacterium was the production of “pseudodiarrhoea” and ejection of *Microcystis* cells through the pedal gape of the mussels. The same authors had already showed in a previously work the increased rejection through the production of pseudofaeces of highly toxic *M. aeruginosa* strain, over low or non-toxic *M. aeruginosa* strains and a non-toxic diatom [38]. In the present study, similar filtration rates were reported concerning the three microalgae studied, the two toxic microalgae producing MC-LR (*M. aeruginosa*) and CYN (*C. ovalisporum*), and the non-toxic microalga *P. keselleri* (Figure 1a) [37]. This result highlights the non-selective grazing behavior of the marine mussel *M. galloprovincialis*. In fact, very little is known about the grazing capacity and selectivity of marine mussels on freshwater microalgae, comparatively to the knowledge gathered about their relatives from freshwaters. Given the present data, we raise the hypothesis that the marine mussel may not be able to recognize these toxic microalgae as a health hazard and/or is less sensitive to the toxicity of these two cyanobacteria. Surprisingly, the marine mussel was also able to feed on the filamentous *C. ovalisporum*, which shows that this species, alongside with *M. aeruginosa*, is a potential health threat and a source of toxins to bivalves. These results, however, should be extrapolated with caution for interpreting environmental scenarios in which colony forming strains predominate, since marine mussels can show selective grazing and rejection of *M. aeruginosa* strains forming large colonies (colony forming *M. aeruginosa* strains were not studied in this experiment).

Bivalve grazing on filamentous cyanobacteria is relatively less known compared to unicellular or colonial cyanobacteria species like *M. aeruginosa*. The invasive golden mussel (*L. fortunei*) showed, in laboratory experiments, similar rates of filtration of single-celled, colonial and filamentous non- toxic cyanobacteria [39]. Nevertheless, whereas single-celled *Microcystis* were ingested, filamentous *Planktothrix* and colonial *Microcystis* were eliminated by mussels in pseudofaeces [39]. Zebra mussels (*D. polymorpha*), on the other hand, showed increased clearance rates of a filamentous and toxic *Planktothrix agardhii* strain, over a non-toxic *P. agardhii* strain and colonial *M. aeruginosa*, in laboratorial grazing experiments [35].

As mentioned before, responses related with feeding on toxic cyanobacteria by bivalve molluscs can range from absent [37] to sublethal and even lethal [40] effects. Indeed, adverse responses include physiological [41], biochemical [42], cellular, and histological [43] effects. In this work two morphological/physiological endpoints, mussel byssus production and dry weight (DW), were analyzed to evaluate the toxicity of the two cyanobacteria species in the marine mussel (Figure 1b,c, respectively). The two endpoints were chosen since they are relatively easy to measure and at the same time can provide useful information regarding the health condition of mussels and growth. DW variations were reported in several cases associated with health impairment and survival. This endpoint was also used to report chronic effects of contaminant exposure. Yet, no variations were observed in these two biological endpoints after 15 days of exposure and feeding on toxic cyanobacteria (Figure 1b,c). Given these results, we advance the hypothesis that this marine mussel is relatively insensitive to these toxic cyanobacteria species and to the toxins MC-LR and CYN, according to the conditions used in this study. In fact, the tolerance shown with regard to *M. aeruginosa* may be related to the low toxicity of the strain used in the study, with an amount of toxin produced of about 0.023 pg/cell. Actually, as mentioned above, also freshwater mussels showed to be insensitive to less toxic *M. aeruginosa* strains, comparatively to highly toxic ones [44]. In this study, marine mussels were also insensitive to *C. ovalisporum*. The strain used from this cyanobacterium, on the other hand, is considered more toxic than *M. aeruginosa* strain since produces greater amount of the toxin CYN (7.854 pg CYN/cell). We cannot discard also the hypothesis that marine mussels rejected and eliminated cyanobacteria cells, after filtration, through pseudofaeces. This defense mechanism would attenuate or reduce the hazard effects of these cyanobacteria. However, this response was not evident in this study.

### 3.2. Mussel Contamination

One of the consequences for bivalves of feeding on toxic cyanobacteria is the accumulation of toxins in the body. The amount of toxin accumulated will depend on several factors associated to toxin intake and the mechanisms of detoxification specific for each species. For instance, in an in situ pond experiment during a *M. aeruginosa* bloom, three freshwater bivalves showed different MCs accumulation in muscle and digestive gland tissues according to grazing, assimilation and detoxification capacities of each mollusc [45]. Nevertheless, most of the studies dealing with cyanotoxins dynamics in bivalves have been done under controlled laboratory conditions with individual cyanobacteria species. Indeed, several studies have shown bivalves’ accumulation of both MC and CYN, generally according with toxin level in phytoplankton. As an example of this, maximum levels of 16.0 µg MCs/g mussel DW were reported by Amorim and Vasconcelos (1999) [46] in an experiment where *M. galloprovincialis* were fed with 10^5^ cells/mL (3.4 µg MC/10^7^ cells) of the toxic cyanobacteria *M. aeruginosa*. The authors also reported the slow and irregular elimination of the toxin from the mussels during depuration which lasted 14 days. Moreover, Amorim and Vasconcelos (1999) [46] reported high amounts of MCs in feces and the release of MCs directly to the water (possibly through urine). This supports the hypothesis that bivalves preferentially excrete, rather than metabolize, these toxins. In a similar study, grazing of freshwater mussel *Anodonta cygnea* on the toxic and filamentous cyanobacterium *Cylindrospermopsis raciborskii* for 16 days resulted in the accumulation of up to 2.52 mg CYN/g tissue DW [13]. Following a two-week depuration, 50% of the toxin remained in the tissues [13]. In this study, with the analysis of toxin content in mussels exposed to a mixture of toxic *M. aeruginosa* and *C. ovalisporum*, we were able to confirm that both MC-LR and CYN were assimilated by mussels and accumulated in the tissues at 14.17 ng MC-LR/g DW and 92.08 ng CYN/g DW (Figure 3). The level of contamination in the above studies is considerably higher than the contamination observed in the present work. Nevertheless, such differences might be attributed to the toxicity of the used phytoplankton species. Also, the used toxic cyanobacteria mixture simulates a common scenario where harmful cyanobacteria blooms often contain a combination of cyanotoxins and results evidence the combined effects from exposure to these (including additive and synergistic effects) which may influence their uptake. Regarding depuration, mussels were effective in the elimination of the majority of CYN content from mussel’s tissues. However, bivalves still presented remnant amounts of both the initial toxins after 15 days of recovery (less than 10%).

### 3.3. Metabolic Responses of Mussels to Toxic Cyanobacteria

Proteomics results evidenced, firstly, that mussels which fed on both cyanobacteria (MIX group) display more differences in their proteome, relatively to the mussels from the other experimental groups (Figure 4). This response may arise from synergistic interplay of cyanobacteria and their secondary metabolites (including cyanotoxins MC-LR and CYN) on mussels. Moreover, the differences observed when comparing proteomic data from the exposition and depuration phases suggest that many proteins undergo changes and return to control expression levels during mussel depuration (Figure 5). This result thus agrees with our expectations with regard to this experimental phase in which the change to a non-toxic diet would provide conditions to mussels recovering from the exposure to the toxic algae.

Overall, the proteomic results point out to a differential response of mussels with respect to its diet (Figure 5 and Figure 6). Feeding on toxic *M. aeruginosa* affected mostly mechanisms related with protein stability and activity in the cells, signal transduction, anion transport and also in the immune response. The over-expression of PDIA, YWHAE and CALR in mussels fed on *M. aeruginosa* (Figure 6a) may be interpreted as a defense mechanism triggered perhaps by the bioactivity of MC-LR or potential interactions of other *M. aeruginosa* bioactive substances with mussel’s proteins, for protecting or stabilize protein activity and function in mussels. PDIA, CALR and YWHAE are involved, respectively, in the following processes/reactions: rearrangement of -S-S- bonds in proteins, protein folding and oligomeric assembly in the ER, and recognition of phosphoserine or phosphothreonine motifs in proteins. Thereby, these proteins play a critical role in the regulation of many other proteins in the cell. Moreover, YWHAE is an adaptor protein and interacts with other proteins in signaling transduction. VDAC2, another protein affected by exposure to *M. aeruginosa*, is a Voltage-dependent anion-selective channel. In the outer membrane of the mitochondria this protein forms a channel that allows diffusion of small hydrophilic molecules. This mechanism is important in the regulation of anion transport and apoptotic signaling. CLC is a galectin. These types of proteins are constituents of the immune system of animals and play a critical role in sensing the presence of pathogenic organisms upon binding to specific β-galactoside sugars in the surface of pathogenic organisms. TLN1 is a cytoskeleton constituent and is believed to be involved in the connection of major cytoskeletal structures to the plasma membrane. A nacre protein was also altered in mussels exposed to *M. aeruginosa*. Nacre proteins are constituents of the shell matrix of bivalves, and among the functions attributed to these proteins is shell calcification [47,48]. Moreover, STRING analysis highlighted the putative role of CALR and YWHAE in mediating the molecular response in mussels, as these proteins seemed to be functionally related with several differentially expressed proteins and showed to be central elements of the protein networks reported (Figure 7a). Feeding on *C. ovalisporum*, on the other hand, seems to affect mainly mechanisms related with ATP synthesis, cell migration and proliferation, protein synthesis and signal transduction/intracellular communication. Among the proteins with altered expression were RGP51, ATP5B, ITIH3, RPL5, nacre protein, and MgC1q12 (Figure 6b). The exposition to these toxic cyanobacteria seems also to induce immune response. Moreover, STRING analysis provided indication that RPL5 and ATP5B are functionally related (Figure 7b). Indeed, RPL5 is a central component of the ribosome, thereby has a major role in ribosomal functions and alongside with protein synthesis. Any alteration in RPL5 and/or ATP5B may thereby affect protein synthesis. RPL5 abundance was repressed in mussels exposed to *C. ovalisporum* (Figure 6b). This molecular effect seems to be related with the bioactivity of CYN and the reported interactions of this toxin with protein synthesis and ribosome activity [49,50]. The functional links between ACTB, ENO1, HSP90, and HNRNPA1, and other differential proteins in mussels exposed to both cyanobacteria, retrieved with STRING analysis (Figure 7c), suggests that these proteins may play a critical role in the response of mussels exposed to both cyanobacteria. The three protein clusters found in this network suggest that the main metabolic processes affected are related with the cytoskeleton (cluster constituted by cytoskeletal and associated proteins, ACTB, MYH7, TPM1, ACTN1, LCP1, FSCN1), protein synthesis (cluster formed by RGN, RPLP2, RPS5, RPN1, HNRNPA1), and other processes involving ENO1 and ACTB (cluster constituted by ACTB, TUBB4B, HSP90AA1, ENO1, CTSD, CTSB, ATP6V1A). Indeed, the high number of protein associations established with ACTB, ENO1 but also with proteins such as HSP90 and HNRNPA1, reported in this functional analysis, suggests that these proteins may play a critical role on the response of mussels and in the majority of the alterations reported in the proteome. ACTB abundance decreased whereas ENO1, HSP90, and HNRNPA1 abundances increased in mussels exposed to both toxic cyanobacteria, pointing to possible disturbances in ATP synthesis and energy metabolism, and the activation of mechanisms related with protein stabilization, protein folding, and activity.

The molecular events that characterize the response of mussels, or other bivalve species, to exposure to toxic cyanobacteria, have been poorly described so far. Among the studies conducted is the one from Puerto et al. (2011) [51], where marine mussels and the freshwater clam *Corbicula fluminea* were fed with toxic cyanobacteria *C. raciborskii*. In this study, changes in cytoskeletal proteins (actin, tubulin isoforms) and also down-regulation of heat shock 60 (HSP60), extrapallial (EP) fluid protein and triosephosphate isomerase (TPI) in gills of *M. galloprovincialis* were observed, which the authors related with tissue injury and physiological stress, caused from exposed to CYN producing *C. raciborskii*. Alongside this, the exposure to toxic cyanobacteria may have affected oxidative stress defense mechanisms involving GPx and GST enzymes.

Other molecular events reported in bivalves, which have been linked to the exposure to toxic *M. aeruginosa* strains include GST transcriptional and proteomic variations. Indeed, GST enzymes have been considered to play a major role in the process of elimination/excretion of MCs, by catalyzing the binding of the substrate glutathione to the toxin. Induction of sigma 1-class GST transcripts was observed in *Ruditapes philippinarum* upon 24-h exposure to toxic *Microcystis* extract. The post-exposure phase was characterized by an early induction (24 h) of sigma 1 and mu transcripts and a later induction (72 h) of the four analyzed GST transcripts (sigma 1, sigma 2, pi, and mu-class GST transcripts) [52]. Also concerning enzymatic activity and protein expression, significant alterations in GST according to bivalve species, were reported. It was denoted in this study a slow response of mussel’s GST system, comparatively to the GST systems from clams *R. philippinarum* and *C. fluminea* [23]. No variations in GST system or other protein markers referred by other authors were observed in the present study. This, on one hand, may reinforce the hypothesis that marine mussels did not undergo major molecular and physiological stress in this study, thereby the poor relation of the molecular results of the present study with others. On the other hand, GST proteins might have remained outside of the present proteomics analysis, as the investigation of this specific group of proteins requires specific proteomics methods which were not employed in the present study. The metabolic response of mussels could, indeed, be related to a process of adaptation of the animal to a new type of diet, and not necessarily to a toxicological outcome, as no evidences of physiological stress were observed in this study that could support such hypothesis. Indeed, differences among experimental groups in terms of the quantity and quality of the diet were likely to occur in this study, related to the size but also the nutritional composition of cyanobacteria and the green alga cells, which can diverge considerably and can have impact in the metabolism and growth of filter feeders [53,54]. These differences were not possible to prevent in the experiment, which was normalized based on the density of microalgae cells. Thereby, such differences may also contribute to the proteomic changes found in mussels. Moreover, cyanobacteria are known to produce a variety of bioactive secondary metabolites [55,56] and their bioactivity, along with the bioactivity of toxins MC and CYN, also may accounted to proteome changes.

On the other hand, the lack of molecular evidence of toxicity suggests that the toxins accumulated in mussels were below the toxic threshold, due possibly to an efficient excretion system of toxins in mussels.

## 4. Conclusions

Contamination of estuarine and coastal ecosystems by toxic cyanobacteria and cyanotoxins has been reported in various locations around the world. This should therefore be considered a real problem, tending to increase in intensity and frequency in different geographical regions due to climate change. On the other hand, impacts of toxic cyanobacteria on marine and estuarine aquatic ecosystems are not as well studied as they are on freshwater ecosystems. This study led to the conclusion that species such as the marine mussel *M. galloprovincialis* can filter and feed on toxic cyanobacteria such as the single celled and colony forming *M. aeruginosa* and the filamentous *C. ovalisporum*, and thereby are potential repositories and vectors of toxins MCs and CYN in marine ecosystems. On the other hand, after 15 days of feeding on toxic cyanobacteria, the marine mussel demonstrated an ability to resist the potential toxicity of this diet. Surprisingly, there were no changes in animal weight, bissus production capacity, or biochemical markers (total protein and glycogen) studied, leading to the hypothesis that the marine mussel has its own mechanisms to defend against cyanotoxins. It has also been shown that mussels are capable to eliminate cyanotoxins such as CYN during the clearance phase of the experiment. This mechanism will have a protective effect on the animal, possibly preventing the accumulation of toxic levels of CYN. Alterations in the animal proteome further suggest that the animal adapt the metabolism in function of the diet. Some molecular responses may be associated with defense mechanisms, such as overexpression of ENO1, HSP90, and HNRNPA1, which could be associated with processes of stabilization of proteins, regulation of protein folding, and activity in cells.

## 5. Materials and Methods

### 5.1. Microalgae Culture

**Figure 8:**
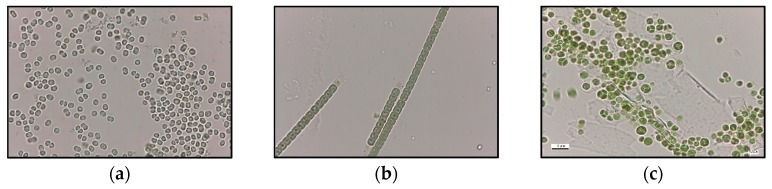
Optical micrographs of *M. aeruginosa* cells (**a**), *C. ovalisporum* cells (**b**) and *P. kessleri* cells (**c**).

The three microalgae species used in this work were obtained from Blue Biotechnology and Ecotoxicology Culture Collection (LEGE-CC) [57]. The microalgae were: *Parachlorella kessleri* (LEGE Z-001), *Microcystis aeruginosa* (LEGE 91094), and *Chrysosporum ovalisporum* (LEGE X-001). These were cultured in Z8 medium, using deionized and autoclaved water as described by Pinheiro et al. (2013) [58]. Cultures were kept, at all stages, in a growth chamber at controlled temperature conditions of 26 °C ± 1 h, with photoperiod of 14 h/10 h (light/dark), and light intensity of 20 μmol m^−2^s^−1^. To secure enough supply of fresh microalgae cells, cultures were established in large 20 L flasks (Thermo Scientific, Waltham, MA, USA) with continuous aeration. *P. kessleri* is a non-toxic green alga whereas *M. aeruginosa* and *C. ovalisporum* are cyanobacteria. *M. aeruginosa* is characterized by small cells (2.6 ± 0.3 μm diameter) and is unicellular whereas *C. ovalisporum* is a filamentous species, with trichomes varying from 0.5–1.0 mm in lenght, narrowing in the extremities, and cells with 4–5 µm diameter (Figure 8a,b, respectively). The strains used are known to produce, respectively, the toxins MC-LR and CYN [57,58,59,60]. Furthermore *P. kesseleri* is a unicellular alga. Cells are spherical with a diameter varying between 1.5 and 10 µm (Figure 8c). Cell density was estimated in a light microscope (Leica, model DM LB 305, Wetzlar, Germany) with a Neubauer chamber. The magnification used was 100× to quantify *P. kessleri* and *M. aeruginosa* cells and 1000× for *C. ovalisporum* cells.

### 5.2. Toxin Extraction and Quantification in Microalgae

Toxins were extracted and quantified from *M. aeruginosa* and *C. ovalisporum* cells as described by Pinheiro et al. (2013) [58]. Briefly *M. aeruginosa* cultures were subjected to sonication in a water bath (RK 100H, Bandelin Sonorex, Berlin, Germany) for 15 min followed by probe sonication in ice, 5 cycles of 1 min at 60 HZ (Vibracell VC50, Sonic & Materials Inc., Newtown, CT, USA). The homogenate was centrifuged (4495× *g*, 4 °C for 10 min) and the supernatant containing the toxin MC-LR was stored at −20 °C. CYN was extracted from *C. ovalisporum* cultures with 0.1% (*v*/*v*) trifluoroacetic acid (TFA) in ultrapure water, homogenized for 30 min, lysed by ultrasound for 15 min at 35 Hz in a water bath, and followed by probe sonication in ice at 20 Hz for 5 min. The homogenate was centrifuged at 4495× *g*, 4 °C for 10 min, and the supernatant fraction were stored at −20 °C. The toxins were quantified by HPLC (Waters Alliance e2695, Milford, CT, USA) equipped with a photodiode array (PDA) detector 2998 (Waters, Sacavém, Portugal). The procedures are described in Pinheiro et al. (2013) [58] and Pereira et al. (2018) [60]. MC-LR was quantified using a calibration curve calculated from a set of seven standard MC-LR dilutions (0.33 to 18 μg/mL MC-LR) in 50% (*v*/*v*) MeOH. The limit of detection (LOD) of this procedure is 0.2 μg/mL MC-LR and the limit of quantification (LOQ) 0.5 μg/mL. A set of seven standard concentrations in a range of 0.30 to 22 μg/mL CYN was used to quantify the CYN from the cultures. The LOD for this procedure was 0.3 μg/mL and LOQ was 0.8 μg/mL.

### 5.3. Mussel Harvest and Maintenance

800 specimens of *M. galloprovincialis* (length 60 ± 15 mm) were harvested at Praia da Memória [46,51], located in the North Coast of Portugal at latitude 41.23041568 and longitude −8.72195363, during low tide. Water temperature in this stretch of coastline vary between 13 °C and 17 °C, and salinity between 30 and 35 ‰. The pH is between 7.5 and 8.5. Mussels were transported in thermal boxes with sea water to the laboratory and cleaned of any algae and other invertebrates attached to their shells.

Mussels were distributed randomly in 8 aquaria filled with 30 L filtered sea water (sand filter and ultra violet), with a total of 93 mussels per aquarium. From those, 40 mussels were used to determine filtration rates, byssus production, dry weight and biochemical parameters, 32 to determine toxins and 16 for proteomics studies, at different time-points (as described bellow). Mussels were maintained in aquaria for 21 days with continuous aeration, temperature was 15 °C ± 2°, salinity 34 ± 2‰, pH 7.90 ± 0.10, dissolved oxygen 8.00 ± 0.50 mg/L, and natural light conditions. During this period animals were fed daily with the green microalga *P. kessleri* (1 × 10^5^ cell/mL) [46]. The water from aquaria was renewed every 2 days.

### 5.4. Experimental Design

**Figure 9:**
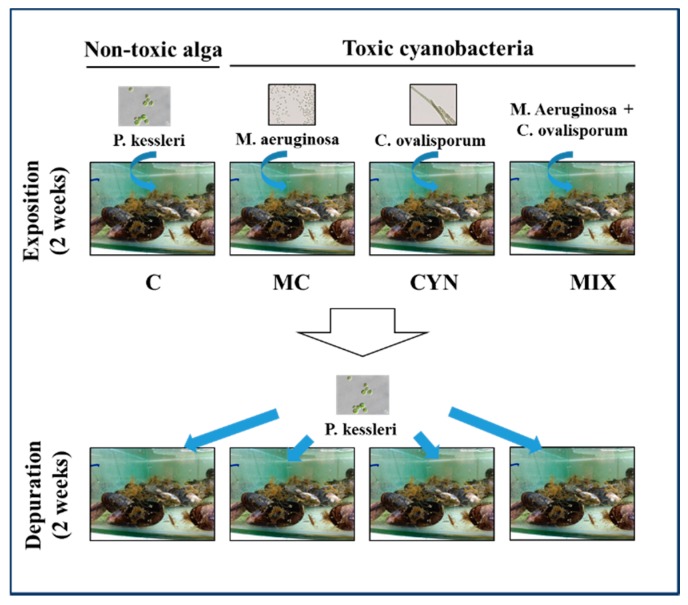
Schematic representation of the exposition and feeding experiment outlined in this wok, to investigate the response of the marine mussel *M. galloprovincialis* to toxic cyanobacteria and its recovery.

After acclimation, an experiment was carried out to simulate a potential natural bloom scenario and mussel’s exposure and feeding on toxic microalgae. Four experimental groups were outlined for this study as shown in Figure 9, a control group (C) in which mussels were fed during the whole study with the non-toxic *P. kessleri* at 1 × 10^5^ cells/mL. Mussels of the other experimental groups were fed, during the first 14 days (exposition phase), separately with the toxic microalgae *M. aeruginosa* (MC group), *C. ovalisporum* (CYN group) or with both cyanobacteria (MIX group) at a cell density of 1 × 10^5^ cells/mL. After the intoxication phase, the toxic microalgae were replaced by the non-toxic *P. kessleri* (1 × 10^5^ cells/mL) and mussels were fed with this microalgae for 15 days (depuration phase). Each experimental condition was replicated in 2 aquaria. Water in aquaria was replaced every 2 days and microalgae was administered twice a day at 1 × 10^5^ cells/mL. The amount of microalgae added to aquaria and the volume of the aquaria were adjusted during the course of the experiment, according to the total mussel load in the aquaria. Sixteen mussels were collected at the beginning of the experiment (T0) and at the end of the first and second weeks of the experiment (T1, T2) that correspond to the period of exposition, and the third and fourth weeks (T3, T4), corresponding to the period of the depuration.

### 5.5. Physiological Parameters

#### 5.5.1. Filtration Rates

Five mussels were collected randomly from each aquarium (10 mussels per experimental group, *n* = 10) before initiating the experiment (T0), and then every week along the 4 weeks of the study (T1, T2, T3 and T4). The bivalves were washed and the visible byssus threads carefully cut. Mussels were placed in individual flasks with 300 mL of seawater and air pumping. The animals were allowed to acclimate for 24 h to the conditions. Then, mussels were fed with 1 × 10^5^ cells/mL of either *P. kessleri* (C), *M. aeruginosa* (MC group), *C. ovalisporum* (CYN group) or a mixture of both cyanobacteria species (MIX group). Water samples were taken every 5 min to count the microalgae cell density, in the microscope with a Neubauer chamber. Mussel’s filtration rates were calculated according to the formula of Jørgensen (1981) [61]:

$$F=V\times \frac{logC_{ic}-logC_{f}}{T}$$

where *F* is the filtration rate of each individual mussel (mL/ind.h), *C_ic_* and *C_f_* are respectively the initial and the final microalga concentrations (cells/mL), *V* is the water volume in the flask (mL), and *T* the time period monitored (min).

#### 5.5.2. Byssus Count

After determining the filtration rate, the number of new byssus produced by the mussels kept in the individual flasks (10 mussels per experimental group, *n* = 10) within the 24 h was visually counted. The animals were then removed from the flasks and used for the calculation of dry weight.

#### 5.5.3. Fresh and Dry Weight

Mussels were collected from the flasks (*n* = 10), the whole body separated from the shelves and the fresh weight measured. The corresponding dry weights were measured after freezing at −80 °C and lyophilization of the tissues for 7 days.

### 5.6. Biochemical Parameters

To perform the quantification of total protein and glycogen, dried tissues from individual mussels (5 mussels per experimental group, *n* = 5) were first turned into powder with a kitchen blender. For total protein quantification 40 mg of tissue powder was mixed with 1 mL phosphate buffer (0.1 M and pH 7.4) and the mixture homogenized by sonication (60 Hz, 3 cycles of 10 sec). The samples were centrifuged for 20 min at 16,000× *g* (4 °C) in a Micro Star 17R (VWR, Radnor, PA, USA) centrifuge. The supernatant was collected and the total protein measured with the Bradford method using a commercial reagent (Bio-Rad), at 495 nm in a microplate reader (Synergy HT, BioTek, Winooski, VT, USA). BSA was used as standard. Total glycogen was quantified from 40 mg tissue powder. The material was mixed with 1 mL phosphate buffer (pH 7.0) with Triton X-100 (0.1% *v*/*v*) and homogenized by sonication (60 Hz, 3 cycles of 10 sec). The supernantants were collected and stored after 10 min centrifugation at 10,000× *g* (4 °C). Total glycogen was determined with a colorimetric method described by Dubois et al. (1951) [62]. Each sample (10 µL) was mixed with 100 µL phenol (5% *v*/*v*) and 600 µL sulfuric acid (100%). The mixture was incubated for 30 min with shaking. Afterwards, absorbance of the mixture was read at 492 nm in a microplate reader. A standard curve was prepared with d-(+)-Glucose (Sigma) in 7 different concentrations ranging from 0.1 to 5.0 mg/mL, for the estimation of total glycogen.

### 5.7. Toxin Analysis in Mussels

MC were extracted from exposed mussels following the method described by Freitas et al., (2014) [63] with slight modifications. Lyophilized mussels (1 g) were extracted with 10 mL of 50% (*v*/*v*) MeOH at room temperature, then stirred in a ultraturrax (1 min), sonicated (10 min), and centrifuged at 4495× *g* at 4 °C for 15 min. The supernatant was collected and the extraction procedure was repeated by adding 10 mL of 50% (*v*/*v*) MeOH and left stirring for 30 min. The two supernatants were pooled together and submitted to a solid phase extraction (SPE) with C18 cartridges (Bakerbond^®^, 500 mg, 6 mL, Dicsa, España). The cartriges were activated with 10 mL MeOH 100% (*v*/*v*) and 10 mL MilliQwater, and after loading the sample, the SPE cartridge was washed with 10 mL of 20% (*v*/*v*) MeOH and MC-LR was eluted with 10 mL of 80% (*v*/*v*) MeOH. Once evaporated to dryness, the residue was resuspended in 1 mL 80% (*v*/*v*) MeOH, and the samples were determined. The extraction of CYN from mussels was performed according to Freitas et al. (2016) [64], followed by SPE from Guzmán-Guillén et al. (2015) [65]. Briefly, lyophilized mussels (1 g) was extracted with 10 mL of 90% (*v*/*v*) acetonitrile (ACN) by sonication (15 min) and centrifuged at 4495 g at 4 °C for 15 min. The supernatant was purified by SPE with graphitized carbon cartridges (BOND ELUT, 500 mg, 6 mL, Agilent Technologies, Amstelveen, The Netherlands). Chromatographic separation for MC-LR or CYN was performed in a UPLC Acquity (Waters) coupled to a Xevo TQ-S micro (Waters) consisting of a triple quadrupole mass spectrometer equipped with an electrospray ion source, operating in positive mode. UPLC analyses were performed on a 100 × 2.1 mm XSelect HSS T3 2.5 µm column, at a flow rate of 0.45 mL min^−1^. A binary gradient consisting of (A) water and (B) ACN, both containing 0.1% formic acid (*v*/*v*) was employed, and injection volume was 5 µL. The elution profile was: 2 % B (0.8 min), linear gradient to 70% B (6.2 min), 100 % B (1 min) and finally 2 % B (2 min). Multiple Reaction Monitoring (MRM) was applied, where the parent ions and fragments ions were monitored at Q1 and Q3, respectively. The mass spectrometer was set to the following optimised tune parameters for UPLC-ESI-MS/MS analyses: capillary voltage to 1.0 kV; source temperature to 500 °C; source desolvation gas flow to 1000 L/h; and source cone gas flow to 50 L h^−1^. The transitions employed for setting up the UPLC-MS/MS system were: 996.5/135.0, 996.5/213.1, and 996.5/996.5 for MC-LR; 416.2/194.0 and 416.2/176.0 for CYN. The first values for each toxin were used for quantification, while the others for confirmation [66].

### 5.8. Proteomics

#### 5.8.1. Sample Preparation

Four mussels were collected per aquarium at each time-point for proteomic studies. Mussels were immediately dissected and the digestive glands isolated. Pools of tissues from 2 animals were made, resulting in 2 replicate samples per aquarium. In total, 4 replicate samples were obtained from 2 replicate aquaria and per experimental group (*n* = 4). The biological material was stored at −80 °C for further processing. In this work only the samples from time-points T2 /end of exposition) and T4 (end of depuration) were processed further. Digestive gland samples (approx. 0.2 g fw) were homogenized in Tris (100 mM), SDS 2% (*w*/*v*), dithiothreitol (0.1 M), pH 7.6 and protease inhibitors (complete protease cocktail tablets, Roche, Basel, Switzerland) with sonication (6 cycles of 5 s at 60 Hz, and incubated overnight. Samples were denatured with heat (95 °C, 3 min) and clarified at 16,000× *g* for 20 min. The supernantants were recovered and total protein estimated at 280 nm. Proteins were digested following the filter aided sample preparation method described by Wisniewski et al. (2009) [67], using centrifugal filter units with nominal molecular weight limit (NMWL) of 30 kDa (MRCF0R030, Millipore, Billerica, MA, USA). Protein samples (40 μg protein) were alkylated with iodoacetamide and digested with trypsin (recombinant, proteomics grade, Roche) for 16 h at 37 °C, at an enzyme to protein ratio of 1:100 (*w*/*w*). Protein digests were recovered by centrifugal filtration, acidified with formic acid (FA) (10%, *v*/*v*), desalted, and concentrated by reversed phase extraction (C18 Tips 100 µl, Thermo scientific, 87784). Before LC–MS/MS, the peptides were recovered in 0.1% (*v*/*v*) FA to the concentration of 0.04–0.06 μg/μl.

#### 5.8.2. LC-MS/MS

The LC-MS/MS was carried out as described previously in a nano-LC coupled to a hybrid Ion trap mass spectrometer (LTQ Orbitrap Velos Pro–ETD, Thermo Scientific, Waltham, MA, USA) [32,68]. Peptides were separated by reverse phase chromatography on a 20 mm × 100 μm C18 precolumn followed by a 100 mm × 75 μm C18 column (particle size 5 μm, NanoSeparations, Nieuwkoop, Netherlands) in a linear gradient of acetonitrile (2% to 95% *v*/*v*) in FA (0.1% *v*/*v*), at a flow rate of 0.3 μL/min (total elution time 70 min). Full scans were performed at 30,000 resolution at a range of 380–2000 *m*/*z*. The top 20 most intense ions were isolated and fragmented with collision induced fragmentation (CID) applying normalized collision energy of 30%, isolation width of 2.0, and activation time of 10 ms and a Q-value of 0.25. In total, 32 independent LC-MS/MS runs were performed, corresponding to the analysis of 32 biological samples.

#### 5.8.3. Protein Identification and Quantification

Proteins were identified by searching LTQ raw data against a custom protein database using SEQUEST algorithm (Proteome Discoverer software, version 1.4, Thermo Scientific, Waltham, MA, USA) and the X!Tandem algorithm in Scaffold (version Scaffold 4.3.4, Proteome Software, Portland, OR, USA) as described previously [32]. Peptides were accepted if established at greater than 95.0% probability by the Scaffold local false discovery rate (FDR) algorithm and proteins were accepted if established at greater than 99.9% probability. MS and MS/MS tolerances were set to 10 ppm and 0.6 Da. Trypsin was selected for protein cleavage allowing for 1 missed cleavage. Carbamidomethylation and oxidation were selected as static and dynamic modifications respectively. The custom database included all sequences of *M. galloprovincialis* transcriptome (46,791 sequences) [32] and the sequences corresponding to the taxa *Mollusca* available from Uniprot (199,262 sequences, released by 04-2016).

Functional analysis of the differential proteins was carried out using the web resource STRING (https://string-db.org/). The analysis was carried out using as reference genes the homologous from Human. Human gene identifiers were retrieved from UNIPROT database (https://www.uniprot.org/) and after searching for Human proteins with names identical to the proteins identified by proteomics. A BLAST search was carried out (E-threshold < 10^−10^) to help to find the corresponding homologous protein in Humans and the respective gene identifier. STRING analysis was performed by selecting *Homo sapiens* database for searching protein interaction evidences. Sources of protein interaction evidences included: textmining, experiments, databases, co-expression, neighborhood, gene fusion, co-occurrence. Other settings considered in the analysis were: Interaction score—medium confidence (0.400) and max number of interactors (1st shell)—no more than 5.

### 5.9. Statistics

Physiological and biochemical parameters were analyzed with linear models performed with R software [69]. Some of the parameters measured (filtration rates, byssus counts, protein content, and glycogen content) were used as dependent variables, being the replicates of each individual measurement taken in a single mussel. Treatment (kind of algae offered) was introduced as independent variable (fixed factor). Dry weight was also included as a covariate, but it was never significant, so it was not considered in the final model. In order to take into account for autocorrelation structure of mussels measured inside each aquarium, ‘aquarium’ was used as random factor. In addition, in order to test for temporal autocorrelation (repeated measurements), ‘time’ was also included as random factor. Different model versions, combining all the possible fixed and random factors together and alone, were fitted, and the best one seleced by the lower AIC (Akaike information criterion). Residual normality and homogeneity of variances were evaluated respectively with the Shapiro–Wilks and Levene tests. Differences between data in treatment × time combinations were analyzed with a Student *t*-test for dependent samples.

Proteomics data was analized with the software Multi Array Viewer version 4_9_0 (mev.tm4.org) using non-parametry tests (Kruskal–Wallis and Mann–Whitney). The significance of the analyses were assessed also at *p* < 0.05.

## Supplementary Materials

The following are available online at https://www.mdpi.com/2072-6651/12/3/196/s1, Table S1: Results of the general linear models performed using the species treatment as fixed factor, Table S2: Results of significant post-hoc Tukey comparisons performed between factor levels in those cases when the factor ‘Filtration rate’ was significant, Table S3: Results of the general linear models performed using the Exposure/Depuration treatment as fixed factor, Table S4: NSAF values and Kruskal-Wallis p-values of all differentially abundant proteins, Table S5: NSAF values and Man Whitney p-values of all differentially abundant proteins.
