# Supplementary material for: Physiological and Metabolic Responses of Marine Mussels Exposed to Toxic Cyanobacteria Microcystis aeruginosa and Chrysosporum ovalisporum
Source: Toxins (Basel). 2020 Mar 20;12(3):196. doi: 10.3390/toxins12030196 (PMC7150937; doi:10.3390/toxins12030196)
Supplement: Supplementary file 1 [file toxins-12-00196-s001.zip › suppl tables/Supplementary tables S1_S3 revised final.docx]

Supplementary Materials: Physiological and Metabolic Responses of Marine Mussels Exposed to Toxic Cyanobacteria *Microcystis aeruginosa* and *Chrysosporum ovalisporum*

Flavio Oliveira, Leticia Diez-Quijada, Maria V. Turkina, João Morais, Aldo Barreiro
Felpeto, Joana Azevedo, Angeles Jos, Ana M. Camean, Vitor Vasconcelos, José Carlos Martins and Alexandre Campos

**Table S1.** Results of the general linear models performed using the species treatment as fixed factor. Chi-squared, df and p-values are referred to the fixed factor.

| **Dependent Variable** | **Significant Random Factors** | **Chi-Squared** | ***df*** | ***p*-value** |
| --- | --- | --- | --- | --- |
| Filtration rate | Time | 11.8 | 3 | <0.01 |
| Byssus | Aquarium | 2.1 | 3 | 0.54 |
| Protein | Time, Aquarium | 2.7 | 3 | 0.44 |
| Glycogen | Time | 6.2 | 3 | 0.1 |

**Table S2.** Results of significant post-hoc Tukey comparisons performed between factor levels in those cases when the factor ‘Filtration rate’ was significant.

| **Dependent Variable** | **Factor Levels Comparison** | **z** | ***p*-value** |
| --- | --- | --- | --- |
| Filtration rate | *Microcystis-Chrysosporum* | -3.4 | < 0.01 |

**Table S3.** Results of the general linear models performed using the Exposure/Depuration treatment as fixed factor. Chi-squared, df and p-values are referred to the fixed factor.

| **Species** | **Dependent Variable** | **Significant Random Factors** | **Chi-Squared** | ***df*** | ***p*-value** |
| --- | --- | --- | --- | --- | --- |
| *P. kessleri* | Protein | Aquarium | 13.8 | 1 | < 0.001 |
|  | Glycogen | Time | 0.7 | 1 | 0.38 |
| *M. aerugiosa* | Protein | Aquarium | 8.7 | 1 | < 0.01 |
|  | Glycogen | Time | 0 | 1 | 0.99 |
| *C. ovalisporum* | Protein | Time | 1.6 | 1 | 0.2 |
|  | Glycogen | Time | 1.8 | 1 | 0.18 |
| Mixture | Protein | Aquarium | 11.1 | 1 | < 0.001 |
|  | Glycogen | Time | 0.001 | 1 | 0.97 |
